# Tenascin-C inactivation impacts lung structure and function beyond lung development

**DOI:** 10.1038/s41598-020-61919-x

**Published:** 2020-03-20

**Authors:** Sandrine Gremlich, Matthias Roth-Kleiner, Lucile Equey, Kleanthis Fytianos, Johannes C. Schittny, Tiziana P. Cremona

**Affiliations:** 1 Clinic of Neonatology, Department woman-mother-child, University Hospital and University of Lausanne, Lausanne, Switzerland; 2 Department of Bio-medical Research, University of Bern, Bern, Switzerland; 3 Division of Pulmonary Medicine, University of Bern, Bern, Switzerland; 4 Institute of Anatomy, University of Bern, Bern, Switzerland

**Keywords:** Genetic models, Cell proliferation

## Abstract

Tenascin-C (TNC) is an extracellular matrix protein expressed at high levels during lung organogenesis. Later, TNC is only transiently *de novo* expressed to orchestrate tissue repair in pathological situations. We previously showed that TNC inactivation affects lung development and thus evaluated here the implications on lung function in newborn/adult mice. Respiratory function parameters were measured in anesthetized and mechanically ventilated wild-type (WT) and TNC-deficient mice at 5 (P5) and 90 (P90) days of age under basal conditions, as well as following high tidal volume (HTV) ventilation. At P5, TNC-deficient mice showed an increased static compliance (Cst) and inspiratory capacity (IC) relative to WT at baseline and throughout HTV. At P90, however, Cst and IC were only elevated at baseline. Control non-ventilated newborn and adult TNC-deficient mice showed similar lung morphology, but less alpha smooth muscle actin (α-SMA) around small airways. SMA + cells were decreased by 50% in adult TNC-deficient lungs and collagen layer thickened around small airways. Increased surfactant protein C (SP-C) and altered TGFβ and TLR4 signaling pathways were also detected. Thus, TNC inactivation-related defects during organogenesis led to persisting functional impairment in adulthood. This might be of interest in the context of pulmonary diseases with thickened airway smooth muscle layer or ventilation heterogeneity, like asthma and COPD.

## Introduction

Tenascin-C (TNC) is a large hexameric protein and the founding member of the tenascin family of extracellular matrix (ECM) proteins^1,2^. TNC binds to cell surface receptors, ECM proteins or, more recently discovered, soluble factors or pathogens to regulate cell adhesion, migration, proliferation and differentiation^1,3–5^. TNC is a complex protein with post-transcriptional (alternative splicing) and post-translational (glycosylation, citrullination, proteolytic processing) regulations giving rise to numerous isoforms having sometimes opposite effects on cell behavior, moreover in a cell-dependent manner^5,6^. Its expression is highly regulated in time and space, with a strong expression during specific morphogenetic events throughout embryogenesis and organogenesis especially in the lung^1,2,5,7^. In the growing lung, it is expressed at the epithelial-mesenchymal interface at the tips of developing airways during branching morphogenesis, and throughout lung mesenchyme during alveolarization^8,9^. In adult tissues, there is very little or no TNC expression, except in small foci of tissues subjected to high tensile stress or in certain stem cell niches^1,8^. Otherwise, TNC is *de novo* re-expressed only transiently in pathological situations like inflammation or tissue damage, or permanently in diseases like cancer or rheumatoid arthritis^1,10,11^.

TNC is highly expressed in many chronic lung diseases like chronic obstructive pulmonary disease (COPD), bronchopulmonary dysplasia (BPD), idiopathic pulmonary fibrosis (IPF), respiratory distress syndrome (RDS) and asthma, where it is even considered as a marker of severity^12–16^. In the 1990’s, two research groups generated TNC-deficient mice: Saga *et al*.^17^ and Forsberg *et al*.^18^. Both animal models showed that TNC inactivation was not lethal and did not induce any reproductive alteration. Studies in TNC knockout (KO) mice showed that lung disease phenotype was largely reduced in the absence of TNC in acute lung injury^19^, in selective epithelial lung injury^20^ or in ovalbumin-induced asthma^21^.

In lung development, we previously showed that TNC inactivation induced a defect in branching morphogenesis (early lung development)^22^ and alveolarization (late lung development)^23^. In early lung development, absence of TNC reduced the number of branches of the developing airways, and in late development, it delayed alveolarization. However, if newborn TNC KO lungs were shown to have an altered morphometry, adult lungs of TNC KO mice appeared morphometrically identical to WT ones at three months of age^22,23^.

In this study, we sought to evaluate the functional consequences of TNC inactivation in the lung, and the potential long-term repercussions of damage observed during lung development. Our results showed that alterations of the respiratory function not only exist at newborn age, but also persist in adulthood. This is associated with modifications of structural proteins like α-SMA or collagen, and an altered expression of transforming growth factor β (TGFβ) and Toll-like receptor 4 (TLR4) signaling pathways intermediates. Together, these results indicate that even if TNC is no more expressed in adult tissues, its inactivation in early stages persistently affects the lung in adults, in a way that could be relevant for pulmonary diseases with increased airway smooth muscle layer.

## Results

### Long TNC isoforms are only expressed in newborn developing lungs

**Figure 1:**
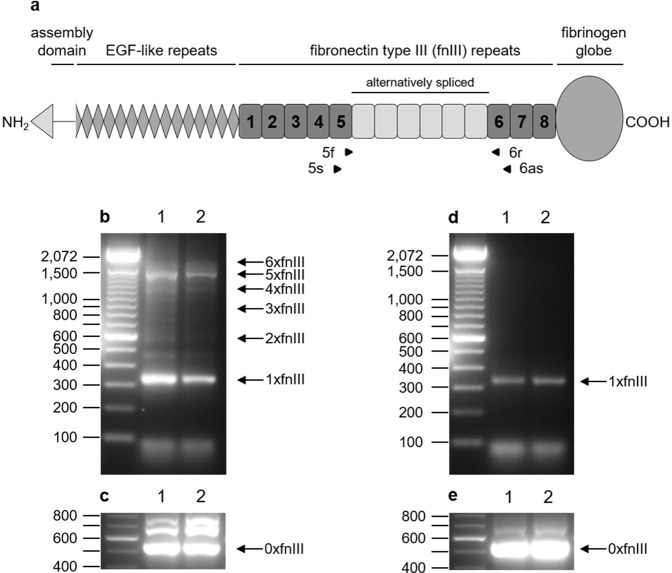
TNC isoforms in newborn and adult WT mice. Lung total RNA (lanes 1–2; two WT samples) was amplified by RT-qPCR with two sets of primers surrounding the alternatively spliced fnIII domains of TNC gene: primers 5f-6r, which immediately border the alternatively spliced region, and primers 5s-6as, located at the 5’ end of fnIII domain 5 and at 3’ end of fnIII domain 6. Panel a: drawing of the mouse TNC gene, with the localization of the primers pairs. Panel b,c: RT-qPCR from newborn lungs; Panel d,e: RT-qPCR from adult lungs; Panel b,d: RT-qPCR with primers 5s-6as; Panel c,e: RT-qCR with primers 5f-6r. Each fnIII domain is 273 nucleotides long. RT-qPCRs are run on 2% agarose gels with 100 bp DNA ladder as size marker. Isoforms are indicated by arrows on the right side of the gel.

**Figure 2:**
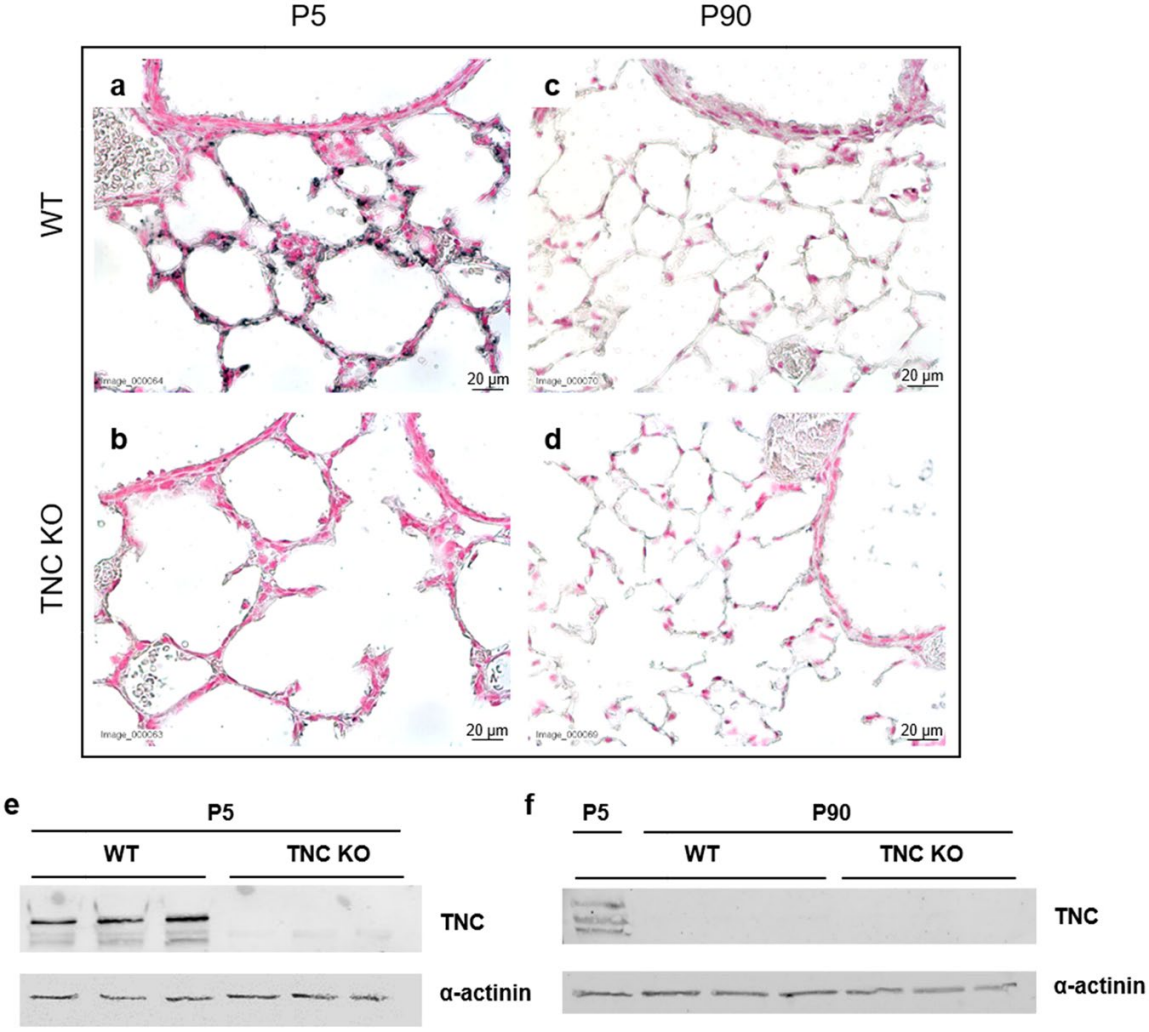
TNC protein expression in lungs of newborn and adult TNC KO and WT mice. Panel a-d: Representative images from lung sections of TNC KO and WT animals, immunostained with anti-TNC antibody (dark grey). Sections were counter-stained with Nuclear Red. (**a**) WT P5; (**b**) TNC KO P5; (**c**) WT P90; (**d**) TNC KO P90. Magnification:40×. N = 4–7 animals for P5, and n = 6 animals for P90. Panel e,f: Representative results of TNC protein detection in whole lung lysates from TNC KO and WT animals by Western blots. Cropped blots are displayed. Full length blots are presented in Supplementary Figure S2. (**e**) newborns lung samples; (**f**) adult lung samples, P5 WT sample as a positive control.

TNC isoforms differ in the number and the composition of alternatively spliced fibronectin-like type III (fnIII) domains present in the mature mRNA, ranging from 0 to 6 fnIII domains in the mouse (Fig. 1a). Primers lining the alternatively spliced region were used to amplify TNC mRNAs by RT-qPCR. In newborns, small and large size PCR fragments were found, with 1–6 alternatively spliced fnIII domains (Fig. 1b,c). In contrast, in adults, only small fragments with 0–1 fnIII domains were observed (Fig. 1d,e). At the protein level, TNC was highly expressed in lung parenchyma in newborn lungs (Fig. 2a,b,e); however, in adult lungs, TNC protein expression was undetectable by immunohistochemistry (Fig. 2c,d) or Western blot (Fig. 2f).

### Basal pulmonary function is persistently altered in TNC KO mice

**Figure 3:**
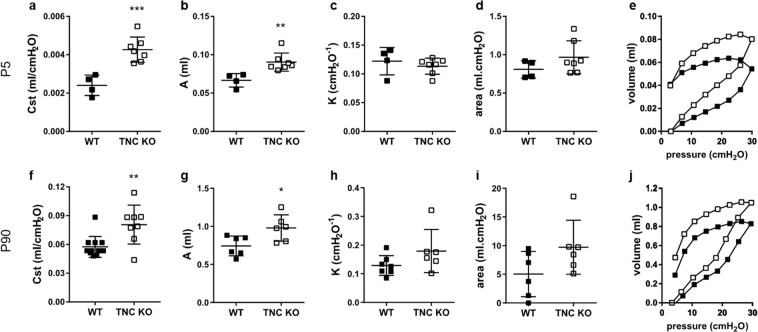
Lung function in newborn and adult TNC KO and WT mice under basal conditions. Respiratory function parameters were collected using a flexiVent system under basal conditions in newborn (P5, Panel a-e) and in adult (P90, Panel f-j) WT (■) and TNC-deficient (□) mice. Panel a,f: static compliance (Cst), describing the distensibility of the respiratory system. Panel b,g: parameter A, estimate of inspiratory capacity. Panel c,h: parameter K, describing the curvature of the PV deflation curve. Panel d,i: hysteresis, estimating atelectasis existing before the PV loop (area between the PV inflation and deflation limbs). Panel e,j: representative PV loops at time 0. P5: N = 4–7 animals/genotype; P90: N = 6 animals/genotype (Cst done on 8–12 animals/genotype). Results are expressed as mean ± SD. Statistical analyses were made by two-way ANOVA; statistical significance was set at p < 0.05; *p < 0.05, **p < 0.01, ***p < 0.001.

**Figure 4:**
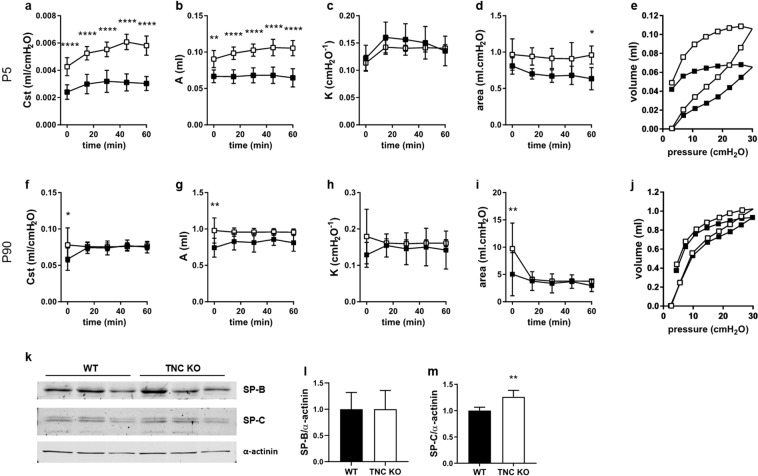
Lung function in newborn and adult TNC KO and WT mice during HTV ventilation. Respiratory function parameters were collected using a flexiVent system during one hour of HTV ventilation in newborn (P5, Panel a-e) and in adult (P90, Panel f-j) WT (■) and TNC-deficient (□) mice. Panel a,f: static compliance (Cst), describing the distensibility of the respiratory system. Panel b,g: parameter A, estimate of inspiratory capacity. Panel c,h: parameter K, describing the curvature of the PV deflation curve. Panel d,i: hysteresis, estimating atelectasis existing before the PV loop (area between the PV inflation and deflation limbs). Panel e,j: representative PV loops at time 60. P5: N = 4–7 animals/genotype; P90: N = 6 animals/genotype. Results are expressed as mean ± SD. Statistical analyses were made by two-way ANOVA; statistical significance was set at p < 0.05; *p < 0.05, **p < 0.01, ****p < 0.0001. Panel k-m: representative results of SP-B and SP-C protein detection in whole lung lysates from adult TNC KO and WT animals by Western blots; quantification is shown in histogram; N = 5 animals/genotype. Cropped blots are displayed. Full length blots are presented in Supplementary Figure S2.

To determine the impact of TNC inactivation on the respiratory function, we measured basal respiratory function in anesthetized and mechanically ventilated newborn (P5) and adult (P90) WT and TNC KO mice (Fig. 3). At P5 and at P90, there were obvious changes in the shape of the PV curves between TNC KO and WT animals (Fig. 3e,j). Relative to WT, static compliance (Cst), an index of the distensibility of the respiratory system, and parameter A, an estimate of the inspiratory capacity (IC), were both significantly higher in TNC KO animals under basal conditions (Fig. 3a,f,b,g). There was no statistical difference in parameter K, a measure of the overall curvature of deflation limb of the PV curves, nor in hysteresis (area), estimating atelectasis existing before the PV loop (Fig. 3c,h,d,i). We also calculated the work of breathing (WOB), which is an indicator of increased effort to breathe, from the area under the inflation limb of the PV curves (Fig. 3e,j). WOB at time 0 was similar in the newborn TNC KO and WT mice at P5 (1.84 ± 0.16 vs 1.86 ± 0.16 J/L, respectively) as well as in the adult mice at P90 (1.72 ± 0.13 vs 1.58 ± 0.31 J/L, respectively, for TNC KO and WT animals). Since TNC expression was observed in tissues exposed to tension, we then investigated TNC KO and WT mice under a mechanical stress induced by a high tidal volume (HTV) profile. Newborn and adult mice were both subjected during one hour to a HTV protocol and respiratory function parameters were recovered every 15 minutes (Fig. 4). Following HTV, differences in the shape of the PV curves between WT and TNC KO animals were only apparent in the newborn mice (Fig. 4e,j). Like what was observed under baseline conditions, static compliance and IC both remained elevated in the TNC KO newborn mice relative to WT during the whole HTV ventilation challenge (Fig. 4a,b). Parameter K was identical in both genotypes during the whole experiment (Fig. 4c) and hysteresis tended to be higher in the newborn TNC KO animals throughout the whole HTV challenge (p ≤ 0.1 at each time point; statistically significant only at the end of the ventilation) (Fig. 4d). In the adult mice, similar PV curves were obtained in the TNC KO and WT mice following 60 minutes of HTV (Fig. 4j), and, consistently, both genotypes showed superimposed values for all related parameters (Fig. 4f–i). In order to test the hypothesis of a change in surfactant production, surfactant protein B and C (SP-B and SP-C) protein expression were evaluated by Western blot. SP-C, but not SP-B, protein expression was slightly increased in adult TNC KO lung samples (Fig. 4k–m).

### Collagen staining is increased around small airways in adult TNC KO lungs

**Figure 5:**
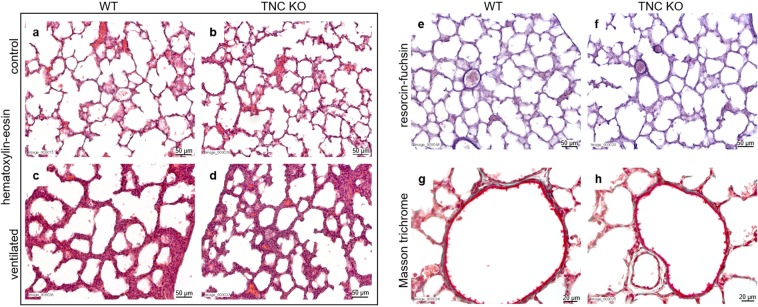
Lung histology in newborn TNC KO and WT mice. Representative images from lung sections of TNC KO and WT P5 animals, either non-ventilated (control) or, for hematoxylin-eosin staining only, ventilated during one hour with HTV ventilation. Five µm lung sections were stained with the following staining: Panel a-d: hematoxylin-eosin staining for morphological studies; Panel e,f: resorcin-fuchsin staining for elastin (dark purple); Panel g,h: Masson Trichrome staining for collagen (green). N = 4–7 animals/genotype. Magnification: 20x for Panel a-f and 40x for Panel g,h.

To evaluate whether certain structures or structural proteins were modified concurrently, histological stainings were performed on P5 lung sections. Hematoxylin-eosin (H&E) staining of lung sections indicated a similar morphology in both TNC KO and WT newborn lungs (Fig. 5a,b). However, when comparing ventilated animals to controls of the same age, we observed a strong thickening and a cellular infiltration of the inter-saccular septa in ventilated animals (both WT and TNC KO) suggesting an inflammatory reaction in response to the HTV ventilation (Fig. 5c,d). Resorcin-fuchsin staining of elastin fibers did not highlight any difference in elastin distribution between TNC KO and WT lungs (Fig. 5e,f). Collagen fibers staining with Masson trichrome showed a faint collagen staining which was not different in TNC KO compared to WT lung (Fig. 5g,h).

**Figure 6:**
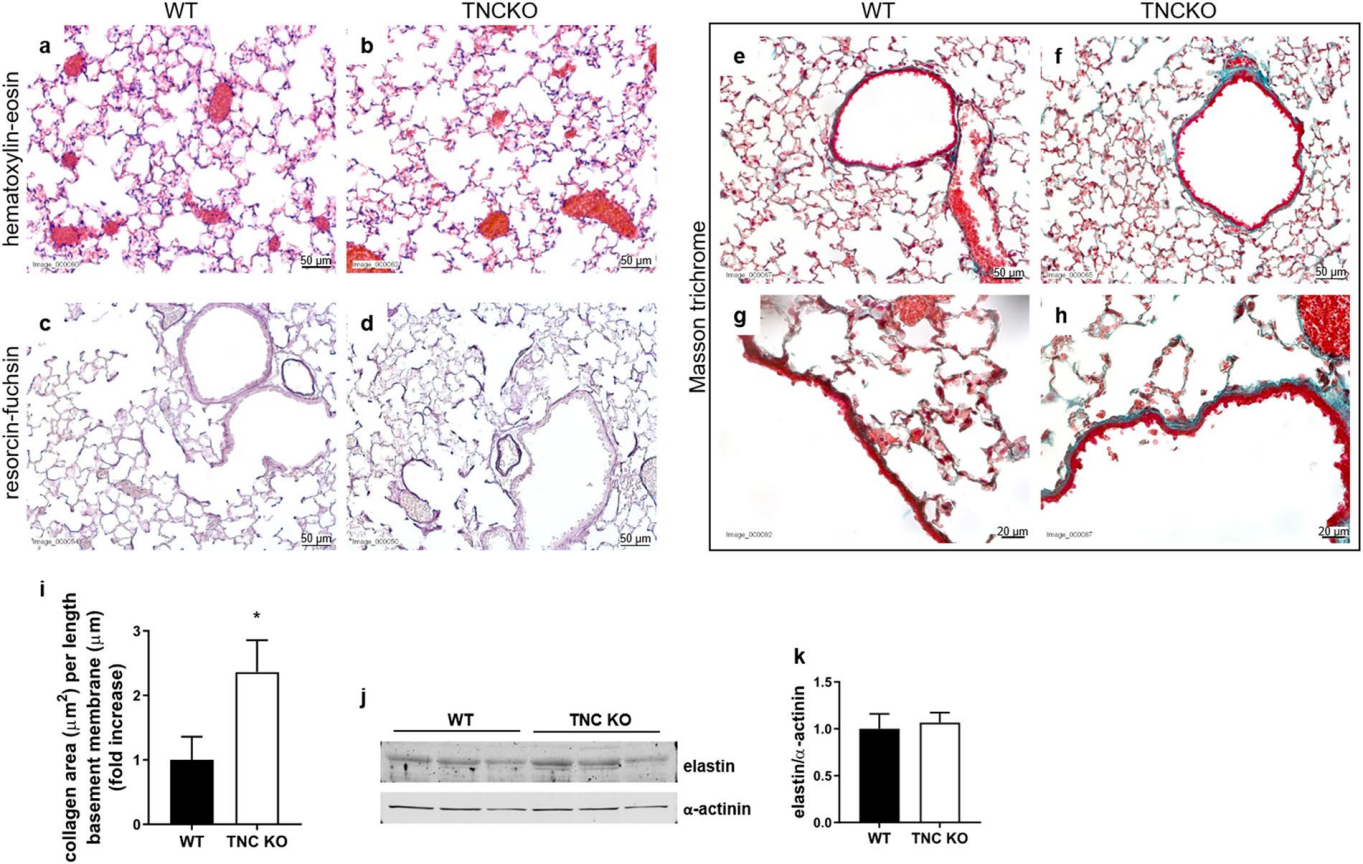
Lung histology in adult TNC KO and WT mice. Representative images from lung sections of TNC KO and WT P90 animals. Five µm lung sections were stained with the following staining: Panel a,b: hematoxylin-eosin staining for morphological studies; Panel c,d: resorcin-fuchsin staining for elastin (dark purple); Panel e-h: Masson Trichrome staining for collagen (green). N = 6 animals/genotype. Magnification: 20x for a-f, and 40x for g,h. Panel i: quantification of collagen area on Masson trichrome staining (40×) using ImageJ; results are expressed as collagen area per length of basement membrane; N = 6 animals/genotype. Panel j,k: representative results of elastin protein detection in whole lung lysates from adult TNC KO and WT animals by Western blots; quantification is shown in histogram; N = 5 animals/genotype. Cropped blots are displayed. Full length blots are presented in Supplementary Figure S2.

The same histological stainings as for newborn (P5) lungs were performed on P90 lung sections. We did not find any morphological differences between TNC KO and WT lungs with hematoxylin-eosin staining (Fig. 6a,b). Elastin staining and protein expression were similar in both TNC KO and WT lungs (Fig. 6c,d,j,k). However, Masson trichrome staining revealed a thicker layer of collagen around small airways in TNC KO animals (Fig. 6e–h). Image analysis confirmed a 2.4 ± 0.5 fold increase in collagen area per length of basement membrane in TNC KO lungs (Fig. 6i).

### Alpha smooth muscle actin (α-SMA) is decreased in newborn and adult TNC KO lungs

**Figure 7:**
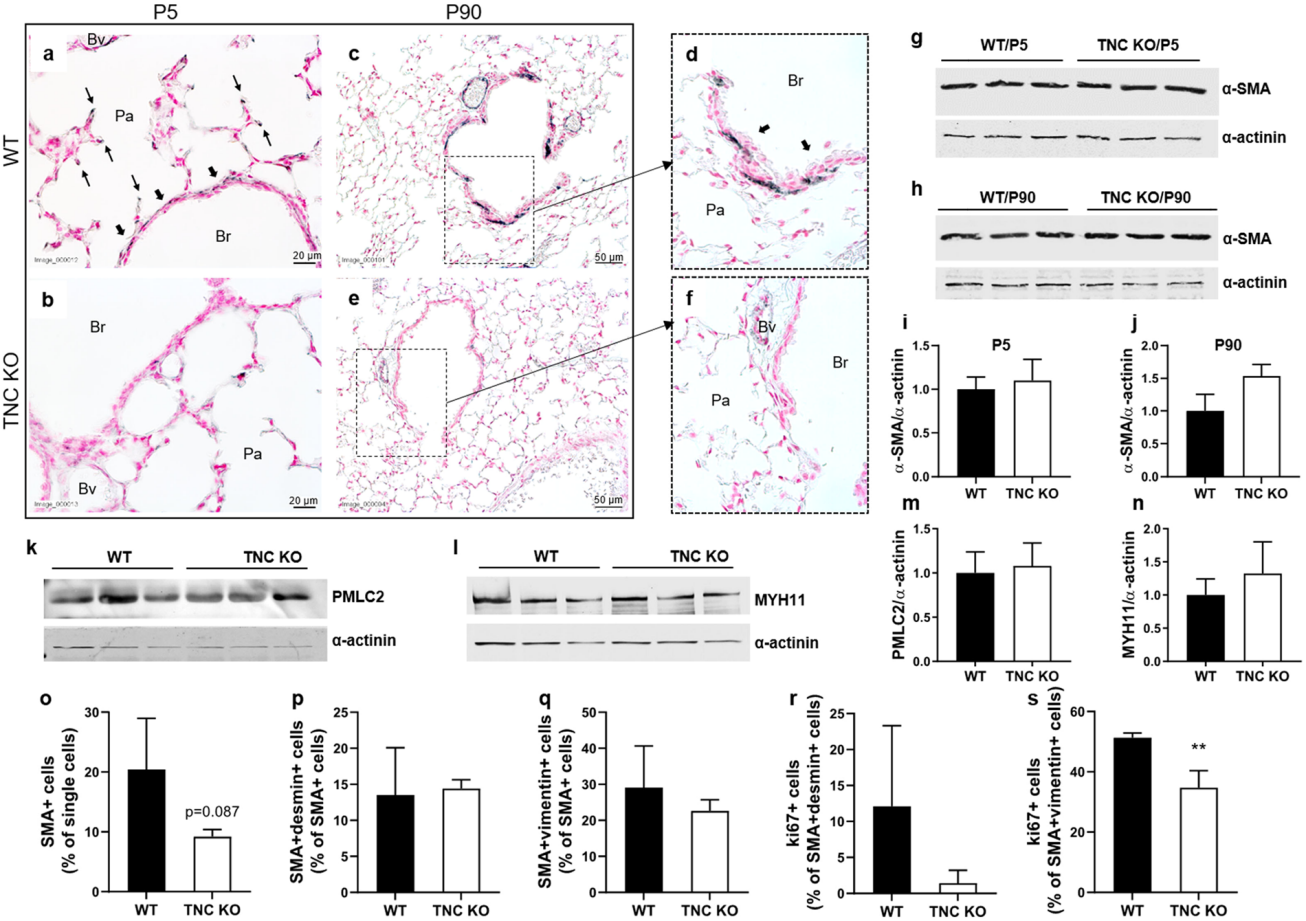
α-SMA protein expression in lungs of adult TNC KO and WT mice. Panel a-f: representative images from lung sections of WT (**a**,**c**,**d**) and TNC KO (**b**,**e**,**f**) P5 (**a**,**b**) and P90 (**c**–**f**) animals immunostained with anti-α-SMA antibody (dark grey), and counter-stained with Nuclear Red. Magnification: 20x for c,e and 40x for a,b,d,f. N = 4–7 animals/genotype for newborns and N = 6 animals/genotype for adults. Panel g-j: representative results of α-SMA protein detection in whole lung lysates from newborn (**g**,**i**) and adult (**h**,**j**) TNC KO and WT animals by Western blots; quantification is shown in histogram; N = 3–4 animals/genotype for newborns and N = 5 animals/genotype for adults. Cropped blots are displayed. Full length blots are presented in Supplementary Figure S2. Panel k,m: representative results of phosphorylated form of smooth muscle myosin light chain (PMLC2) protein detection in whole lung lysates from adult TNC KO and WT animals by Western blots; quantification is shown in histogram; N = 5 animals/genotype. Cropped blots are displayed. Full length blots are presented in Supplementary Figure S2. Panel l,n: representative results of smooth muscle myosin heavy chain (MYH11) protein detection in whole lung lysates from adult TNC KO and WT animals by Western blots; quantification is shown in histogram; N = 5 animals/genotype. Cropped blots are displayed. Full length blots are presented in Supplementary Figure S2. Results are expressed as mean ± SD. Panel o-s: flow cytometry experiment done on adult TNC KO and WT lung samples using α-SMA, desmin and vimentin antibodies. Ki67 was used to measure proliferation. N = 3 animals/genotype. Statistical analyses were made by Student t test; statistical significance was set at p < 0.05; **p < 0.01.

Alpha-smooth muscle actin (α-SMA) protein immunostaining at P5 was weaker in TNC KO lungs, especially around small airways and at the tips of the developing septa, but also to a lesser degree around large airways and arterial blood vessels (Fig. 7a,b). Nevertheless, whole lung α-SMA protein expression was identical in both genotypes (Fig. 7g,i). At P90, α-SMA immunostaining was largely weaker in TNC KO lung around small airways and to a lesser extend around larger airways and arterial blood vessels (Fig. 7c–f). Moreover, flow cytometry experiments showed a 50% decrease (p = 0.087) in the percentage of SMA + cells in TNC KO lungs (Fig. 7o), with a similar percentage of decrease in both genotypes, in SMA + desmin+ cells (smooth muscle cells) and SMA + vimentin+ cells (myofibroblasts) (Fig. 7p,q). Interestingly, the percentage of proliferating cells (ki67 +) of both populations was even lower in TNC KO samples, compared to the same population in WT (Fig. 7r,s). Whole lung α-SMA protein expression was, however, overall slightly increased in TNC KO animals (Fig. 7h,j). In order to test other major proteins of smooth muscle actin fibers, whole lung protein expression of smooth muscle myosin heavy chain (MYH11) and phosphorylated form of smooth muscle myosin light chain (PMLC2) was checked in parallel. Expression of both proteins were the same in both adult TNC KO and WT animals (Fig. 7k–n).

### TNC-related TGFβ and TLR4 pathways are upregulated in adult TNC KO lungs

**Figure 8:**
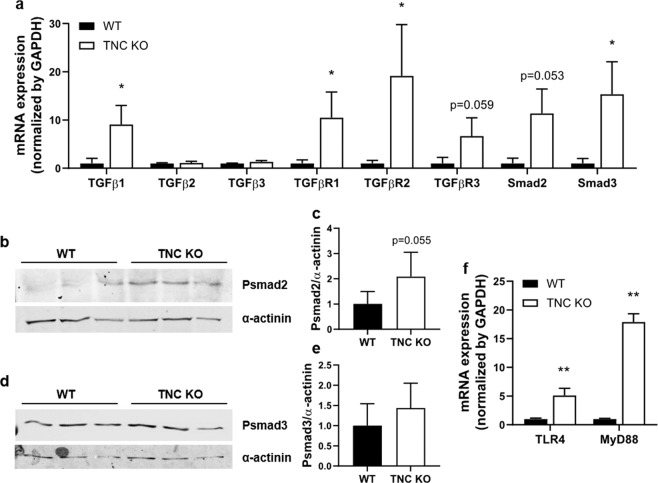
TGFβ and TLR4 pathway intermediates are upregulated in adult TNC KO lungs. Panel a: mRNA expression of TGFβ pathway intermediates evaluated by RT-qPCR in whole lung lobe homogenates of adult TNC KO and WT mice. N = 3–4 animals/genotype. Panel b-e: Representative results of phosphorylated smad2 and smad3 protein detection in whole lung lysates from adult TNC KO and WT mice; 20 or 160ug of whole lung lobe lysates were loaded on 7.5% SDS-Page gels. N = 5 animals/genotype. Cropped blots are displayed. Full length blots are presented in Supplementary Figure S2. Panel f: mRNA expression of TLR4 pathway intermediates evaluated by RT-qPCR in whole lung lobe homogenates of TNC KO and WT adult mice. N = 3–4 animals/genotype. Results are expressed as mean ± SD. Statistical analyses were made by Student t test; statistical significance was set at p < 0.05; *p < 0.05, **p < 0.01.

Expression of transforming growth factor beta (TGFβ) pathway components was evaluated by RT-qPCR in P90 lungs to see if the principal TNC-related pathway was affected in parallel to lung function in TNC KO lungs. TGFβ1 and TGFβ receptor 1 (TGFβR1), -R2 and -R3 mRNA expression were largely increased in TNC KO lungs, whereas TGFβ2 and −3 were unchanged (Fig. 8a). Smad2 and Smad3 mRNAs were also largely increased in TNC KO lungs, but the expression of their phosphorylated protein was only slightly increased, as seen by Western blot (Fig. 8b–e). TNC was also shown to be a ligand for Toll-like receptor 4 (TLR4) and activate the myeloid differentiation primary response 88 (Myd88)-dependent branch of the TLR4 pathway^24,25^. We looked at the expression of two intermediates of this pathway. TLR4 and MyD88 mRNA expression were also highly up-regulated in TNC KO lungs (Fig. 8f).

## Discussion

Based on the previously described critical role of TNC in lung development, we hereby explored how TNC-deficiency affected lung function in newborn mice and whether this had repercussions persisting in adulthood. The aim of this study was thus to evaluate homozygous TNC KO versus WT mice at two different time points: newborn (P5) and adult age (P90 (3 months)). Animals were tested for their lung function under basal conditions and after a mechanical stress challenge in order to highlight functional alterations that may otherwise be silent.

It is well known that different domains compose TNC protein and control its interaction with numerous binding partners^4,26^. Mouse *TNC* gene possesses 8 constant and 6 alternatively spliced fnIII domains, each corresponding to an exon^2,6^. Our results confirmed that whereas mRNAs of short and long isoforms of TNC were found in newborn lungs, only short isoforms were detected in adult lungs. This is in agreement with the collection of studies made in different species and recently published as a review^6^. Short TNC isoforms promote cell attachment, cell differentiation and the formation of focal adhesions, whereas long TNC isoforms promote cell migration and are expressed when morphogenesis is very active, like in embryogenesis or cancer growth^6,8^. At the protein level, we also confirmed that TNC was highly expressed in the parenchyma but not in the conducting airways in newborn WT lung. However, in adult lungs, TNC protein was undetectable, in accordance with previously published reports^27^.

Functional evaluation of the newborn TNC KO and WT mice showed that static compliance and inspiratory capacity were elevated in the TNC KO animals under basal conditions, indicating a higher respiratory system distensibility than their WT counterpart. This difference persisted during one hour of mechanical stress. Interestingly, no alteration was observed in the general lung morphology of TNC KO animals relative to WT. As assessed, static compliance reflects the intrinsic elastic properties of the respiratory system including surface tension. Two ECM proteins are mainly responsible for elastic properties of the lung: elastin and collagen. Collagen represents 50% of lung ECM and is principally composed of fibrillar collagen (collagen I and III). Elastin represents 18% of lung ECM and is made essentially of crosslinked elastin surrounded by fibrillin microfibrils^28^. No difference in elastin or collagen staining was observed between TNC KO and WT newborn lungs. Surface tension depends on the size of airspaces and the amount and distribution of surfactant. We did not detect any morphological difference between TNC KO and WT lungs in hematoxylin-eosin staining, but morphometric measurements done by Mund and Schittny in TNC KO lungs showed the existence of a larger lung volume at P2–21, accompanied by a smaller length of the free septal edge (length of alveolar entrance rings), a reduced formation (anlage) of new alveolar septa, and a reduced alveolar surface area at P7 (as compared to WT); this result was indicative of a delay in classical alveolarization, which was further shown to be caught up during continued alveolarization^23^. Regarding surfactant, no implication of TNC in surfactant metabolism has been shown so far and surfactant protein C (SP-C) immunostaining did not reveal any difference between TNC KO and WT lung sections (data not shown).

Alpha-SMA is a marker of airway smooth muscle (ASM) cells, vascular smooth muscle cells and myofibroblasts in the lung. ASM is the contractile element responsible for bronchoconstriction, airway luminal diameter and airway resistance. ASM also produces ECM proteins, pro- and anti-inflammatory mediators and growth factors necessary to influence the proliferation, migration and apoptosis of other resident cells like epithelial cells, fibroblasts, or ASM cells themselves^29–31^. In newborn TNC KO mice, we observed a weaker α-SMA staining around small airways and at the tips of the developing septa, and to a lesser extent around large airways and blood vessels. It is not clear whether ASM cell layer is thinner or α-SMA is less expressed in ASM cells in TNC KO animals. However, this could potentially be associated with a more heterogeneous ventilation in the TNC KO mice as a result of more easily collapsible small airways. This might also provide an explanation to the fact that compliance remained elevated in the newborn mice following the mechanical stress challenge as the weaker α-SMA staining found at the tips of the free septa (most probably myofibroblasts), representing a reduced α-SMA content in the alveolar entrance rings, would also be expected to result in a decreased tissue resistance and more airway instability during HTV. Overall, the differences in morphometric measurements showing an emphysematous profile of TNC KO lungs compared to WT lungs^23^, and the reduced branching observed in TNC KO lungs explants during early lung development^22^ could explain the increased static compliance and inspiratory capacity observed in newborn TNC-deficient mice.

Adult TNC KO mice, like the newborn ones, also displayed under basal conditions a higher static compliance and inspiratory capacity than their age-matched WT controls, indicating a persistent functional impairment following TNC inactivation. However, under HTV ventilation, both genotypes behaved in a similar manner suggesting that despite its underlying functional impairment, the adult TNC KO lungs were able to cope during the mechanical stress challenge. Adult mice have been previously shown to be more susceptible to HTV mechanical ventilation than newborn mice^32^. Therefore, when newborn mice were ventilated with a tidal volume of 40 ml/kg, adult mice were ventilated with 25 ml/kg for a similar challenge. No striking differences in lung morphology or elastin staining/protein expression were either observed between adult TNC KO and WT mice. Morphometric measurements done previously by Mund and Schittny also showed a similar lung volume, length of the free septal edge, formation (anlage) of new septa and alveolar surface area in both genotypes at 3 months of age (P86)^23^. No difference in SP-C immunostaining was either observed in TNC KO versus WT lung sections (data not shown), but a slight increase in SP-C, but not SP-B, protein expression, speaking for an enhanced efficacy of surfactant, was observed in whole lung lysates from adult TNC KO mice relative to WT. On the other hand, there was clearly a weaker α-SMA immunostaining around small airways and to a lesser extend around large airways and blood vessels as in the newborn mice and, interestingly, collagen staining revealed a thickened subepithelial collagen layer around small airways in TNC KO lungs. These differences in collagen and surfactant might have protected the adult TNC KO lungs during HTV, which is susceptible to induce lung injury as a result of repetitive overdistention and derecruitment. These differences might therefore explain the observed unchanged static compliance in TNC-deficient mice at P90 relative to WT and the lack of a similar effect at P5. Finally, the slight increase in SP-C could also have contributed in the increase in static compliance and inspiratory capacity seen under basal conditions in the adult TNC KO mice.

In adult lungs, flow cytometry experiments confirmed the large decrease in SMA + cells observed in TNC KO lungs by immunohistochemistry. Both smooth muscle cells (SMC) and myofibroblasts cell numbers were decreased. Moreover, the proliferating fractions of these two populations were also lower in TNC KO compared to WT lungs, pointing to a general decrease in SMA + cells proliferation in the absence of TNC. SMA + cells originate from mesenchymal cells during branching morphogenesis (peribronchiolar stage) for smooth muscle cells, and during alveolar stage for myofibroblasts. During branching morphogenesis, pluripotent mesenchymal cells present at the distal end of each growing branch migrate in a distal to proximal direction along the branches. Under the combined action of factors from mesodermal and epithelial origin acting in a cooperative way, they give rise to pools of SMC progenitors and then differentiated SMCs. These factors are principally: fibroblast growth factor 10/9 (FGF10/9), Wnts, sonic hedgehog (Shh), bone morphogenetic protein 4 (Bmp4), platelet-derived growth factor receptor (PDGFR), but also vascular endothelial growth factor (VEGF) or TGFβ^33,34^. Wnt secreted by the adjacent developing epithelium is necessary for expansion and differentiation of SMC progenitors, and it was shown to act through a Wnt/TNC/PDGFR pathway^35^. This could indicate that absence of TNC impairs SMC progenitors pool formation, and therefore, explains the lower number of SMA + cells in TNC KO adult lungs.

At the molecular level though, whole lung α-SMA, but also myosin heavy and light (phosphorylated form) chain protein expression was either slightly upregulated or unchanged in adult TNC KO lungs. It is possible that the discrepancy between α-SMA immunohistochemistry/flow cytometry and Western blot results comes from the monomer versus polymer status of α-SMA protein. Indeed, actin monomers polymerize in actin filaments (with troponin and tropomyosin), and complexes with myosin filaments to form smooth muscle fibers. Alpha-SMA monomers could be detected by Western blot (denaturing gels), but not be uncovered by immunohistochemistry or flow cytometry. Or it could simply be that each SMA + cell expresses higher levels of SMA in TNC KO than in WT lungs. SMA gene expression is under the control of many factors among which TNC, through the TLR4/Myd88 pathway. Indeed, TNC was shown to induce the synthesis of inflammatory markers through the Myeloid differentiation primary response 88 (MyD88)-dependent signaling pathway of TLR4^24,25^. But TNC also induces α-SMA, collagen I and TGFβ expression, as well as myofibroblast differentiation/recruitment, through TLR4 receptor, while in return, TGFβ induces TNC through the TGFβ pathway^13,36–44^. In our experiments, TGFβ pathway intermediates were not down-regulated in TNC KO lungs, but rather up-regulated, maybe as a compensatory mechanism. mRNA expression of TGFβ1 and of all three TGFβ receptors were increased, while TGFb2 and −3 mRNA expression were unchanged. This is in accordance with the fact that TGFβ1 is expressed both during embryogenesis and adulthood, whereas TGFβ2 and −3 are abundantly expressed essentially in developing organs^45^. TLR4 pathway members Myd88 and TLR4 were up-regulated as well in TNC KO lungs. This could contribute to the increase in SMA gene and protein expression. Interestingly, TNC was shown to bind TLR4 via its C-terminal fibrinogen-like globe (FBG) domain. Yet, TNC is not the only protein bearing an FBG domain. Tenascins belong to the fibrinogen-related proteins (FREPs) family that all bear an FBG domain. If not all FBG domains are able to activate TLR4, Zuliani-Alvarez *et al*. showed that the presence of a cationic ridge in the FBG domain defined the capacity to activate TLR4^46^.

Increased TNC expression was reported in lungs of patients with asthma and chronic lung inflammation^10,47,48^. Increased TNC expression was also found in lungs of COPD patients, where an obstructive component also occurs in form of chronic bronchitis^15^. In animal experiments, Nakahara *et al*. showed that TNC inactivation was protective against ovalbumin-induced asthma, with less eosinophils in the bronchoalveolar lavage fluid (BALF), decreased lung cell infiltration and reduced Th2 cytokines IL-5 and IL-13 in BALF and plasma^21^. Doan *et al*. further showed that TNC KO lungs exhibited a decreased expansion while accelerated maturation of eosinophil progenitors in the same asthma model^49^. In a model of bleomycin-induced acute lung injury (ALI), Carey *et al*. showed that TNC KO lungs were protected from fibrosis, with less SMA + myofibroblasts in the lung, and TNC inactivation prevented constitutively active TGFβ to induce the differentiation of fibroblasts into myofibroblasts^19^. ASM hypertrophy is largely implicated in diseases like asthma, but also bronchitis and COPD, where thickening of ASM and airway remodeling lead to a decrease in bronchial diameter or airway obstruction^50,51^. The effect of down-regulation of α-SMA + cells following TNC inactivation could therefore constitute an interesting approach in the prevention or treatment of obstructive pulmonary diseases with thickened ASM layer or heterogeneous ventilation. Indeed, the same Wnt/TNC/PDGFR pathway regulating SMC formation and differentiation during lung development was recently shown to recur in airway remodeling in chronic asthma^52,53^.

Altogether, the results hereby presented demonstrate that TNC deficiency induces defects in lung developmental processes having functional implications that persist in adulthood. TNC being no more expressed at this stage, the functional alteration of adult TNC KO mice probably originated from lung development alterations, but subsisted in adulthood in part due to persistent architectural/structural modifications (SMA + cells, collagen) and impaired gene expression (TGFβ and TLR4 pathways). Silencing of TNC expression might be an interesting approach for the prevention of ASM thickening in patients with functional and structural airway obstruction or ventilation heterogeneity, like in asthma or COPD.

## Methods

### Animals

All experiments were approved by the animal ethic commissions of the Federal Food Safety and Veterinary Office, and the Veterinary Service of the Canton Bern and performed in accordance with the Swiss Federal Act in Animal protection guidelines and regulations. TNC KO mice^18^ were bred on a 129SV background. Postnatal day 5 (P5) and 3 months old (P90) TNC KO and WT animals were anesthetized with a mixture of Midazolam (5 mg/kg BW), Fentanyl (0.05 mg/kg BW) and Medetomidin (0.5 mg/kg BW), and then intratracheally intubated and connected to a computer-controlled piston ventilator (flexiVent, SCIREQ Scientific Respiratory Equipment Inc., Montreal, Canada) for mechanical ventilation and for the construction of pressure-driven pressure-volume (PV) curves. The animals were first ventilated with a standard mouse ventilation profile (respiratory rate: 150/min; tidal volume: 10 ml/kg; PEEP: 3cmH_2_O) and PV curves were constructed in a step-wise manner under closed-chest conditions (basal conditions). Then, mechanical ventilation was switched to a high amplitude ventilation mode (HTV) and the subjects were ventilated for 1 h with room air at a respiratory rate of 60/min, an increased tidal volume (25 ml/kg for the adults and 40 ml/kg for the newborns) and a PEEP of 0cmH_2_O. During that time, PV curves were recorded every 15 minutes, while the animals were kept on a heating pad with rectal temperature being maintained at 35–37 °C and heart rate monitored by ECG. The Salazar-Knowles equation^54^ was fitted to the pressure and volume signals at each plateau of the deflation limb of the PV curves directly in the flexiVent operating system (flexiWare version 7.2) and the following parameters were automatically obtained after each measurement: Static compliance (Cst): describing the distensibility of the respiratory system (lungs and chest walls) at 5 cmH_2_O, parameter A: an estimate of the inspiratory capacity and parameter K: the exponential function capturing the curvature of the PV curves. In addition, the area between the inflation and deflation limbs of the PV curve (hysteresis) was calculated, as well as the work-of-breathing (WOB) from the area under the inflation limb, normalized to the maximal volume.

At the end of an experiment, the animal was sacrificed with an overdose of the anesthetic mixture. The left lung lobe was ligated and snap-frozen for RNA/protein extraction. The right lobes were inflated with freshly prepared paraformaldehyde 4% at a pressure of 20 cmH_2_O, fixed and stored for histological analyses. Pressure was maintained during fixation for at least 2 hours at 4 °C, to prevent the lung from recoiling. Non-ventilated age-matched animals were used as controls.

### RT-qPCR

Frozen lungs were ground to powder using pre-cooled mortar and pestle, and re-suspended in RNAzol (Sigma-Aldrich, Darmstadt, Germany). Total RNA was extracted following the manufacturer’s protocol. Reverse transcription was realized using PrimeScript 1^st^ strand cDNA synthesis kit (Takara Bio Inc., Kusatsu, Shiga, Japan). qPCR was performed using a Corbett Rotor-Gene 6000 apparatus and Rotor-Gene SYBR Green PCR kit (QIAGEN, Hilden, Germany). Primers were: 5s-6as and 5f-6r^55^. Program was: 45 cycles of 20 sec at 95 °C, 30 sec at 57 °C for 5s-6as/59 °C for 5f-6r and 60 sec at 72 °C. qPCR samples were run on 2% agarose gels containing GelRed Nucleic Acid Gel Stain (Biotium Inc., Fremont, CA, USA) and visualized on a UV table. For mRNA expression analyses, primers used are shown in Supplementary Table SI. Program was a standard 2-steps program with 40 cycles of 5 sec denaturation at 95 °C and 10 sec annealing/amplification at 60 °C.

### Histology

Lungs were fixed in freshly prepared 4% paraformaldehyde at 4 °C for 2 h. Fixed lungs were then embedded in paraffin and 5 µm sections were cut using a MicroM microtome (Thermo Fischer Scientific Inc., Reinach, Switzerland). Hematoxylin-eosin (1 min in hematoxylin, 5 min in running tap water, 20 dips in ascending EtOH solutions from 70 to 100%, 20 sec in eosin), resorcin-fuchsin (30 min in Weigert’s resorcin-fuchsin solution, 20 dips in running tap water, 10 sec in acid alcohol, 2 min in tap water, 20 dips in distilled water) and Masson trichrome (1 min in hematoxylin, 5 min in running tap water, 10 min in fuchsin-ponceau solution (1% fuchsin, 1% Ponceau in 1% acetic acid), 20 dips in distilled water, 3 min in 1% phosphomolybdic acid, 1 min in fast green FCF (in 1% acetic acid), rinsing in 1% acetic acid) stainings were used to evaluate basic morphology, elastin and collagen distribution, respectively. Three pictures of two different lung sections of each animal were evaluated by three different examiners (SG, TPC and JCS) for each staining. Criteria for the evaluation of differences between genotypes were to be able to sort the pictures by genotype, not knowing to which animal it belonged. For Masson trichrome staining, a quantitative analysis of collagen was performed by a single examiner (SG) using the free ImageJ software (Rasband, W.S., ImageJ, U. S. National Institutes of Health, Bethesda, Maryland, USA, https://imagej.nih.gov/ij/, 1997–2018). For each high magnification image (40×), a color threshold was applied to identify the stained structure. The results are reported as the collagen area per length of basement membrane.

### Immunohistochemistry

Five µm sections were deparaffinized, treated with 0.3% H_2_O_2_ to block endogenous peroxidases, and rehydrated. Depending on the protein targeted, sections were treated 20 min in 0.01 M sodium citrate pH6 at 95 °C for heat-induced antigen retrieval, before being blocked with pre-immune serum and incubated with primary antibodies for 1–4 hours. Secondary antibody system was composed of biotinylated secondary antibody, Vectastain ABC HRP kit (PK-4000) and Vector SG HRP substrate kit (SK-4700) (Vector Laboratories, Burlingame, CA, USA). Counter-staining was done with Nuclear Fast Red (#6070-5 G) (Fluka, Buchs, Switzerland). Primary antibodies were: anti-α-SMA (A2547) (Sigma-Aldrich, Darmstadt, Germany), anti-tenascin C (LS-C313183) (LifeSpan BioSciences Inc., Seattle, WA, USA). For α-SMA antibody, the Mouse on Mouse Detection Kit was used (BMK-2202) (Vector Laboratories, Burlingame, CA, USA). Pictures of three different experiments were evaluated by three different examiners (SG, TPC and JCS) for each immunohistochemical staining. Criteria for the evaluation of differences between genotypes were to be able to sort the pictures by genotype, not knowing to which animal it belonged.

### Western blot

Frozen lung lobes were ground to powder using pre-cooled mortar and pestle, and resuspended in RIPA buffer (Pierce #89900, Thermo Fisher Scientific Inc., Waltham, MA, USA) plus anti-proteases and anti-phosphatases. Protein lysates were quantified by BCA protein assay (Pierce #23225, Thermo Fisher Scientific Inc., Waltham, MA, USA). Twenty ug (except Psmad2: 160 ug) were loaded on 7.5% SDS-PAGE gels and transferred to nitrocellulose membranes. After blocking with casein 1x solution (#SP-5020 Vector Laboratories, Burlingame, CA, USA) or 5% BSA fraction V (#A1391,0100; Applichem GmbH, Darmstadt, Germany) in TBS 1X (50 mM Tris-HCl, pH 7.5; 150 mM NaCl) for phospho-proteins, membranes were incubated overnight with the following primary antibodies: anti-α-SMA (#A2547) (1:250) and anti-α-actinin (#A5044) (1:250) (Sigma-Aldrich Inc., Saint-Louis, MI, USA), anti-Psmad2 (#3108 S) (1:1’000) and anti-phospho-myosin light chain 2 (#3674) (1:1’000) (Cell Signaling Technology, Danvers, MA, USA), anti-Psmad3 (#ab51177) (1:2’000), anti-elastin (#ab21610) (1:200) and anti-tenascin-C (#AB108930) (1:1’000) (Abcam, Cambridge, UK), anti-SP-C (#sc-13979) (1:100) (Santa Cruz Biotechnology Inc., Dallas, TX, USA), anti-SP-B (#NBP1-57977) (1:500) (Novus Biologicals, Centennial, CO, USA) and anti-myosin heavy chain 11 (#GTX131414) (1:500) (Genetex Inc., Irvine, CA, USA). They were subsequently incubated with the following secondary antibodies during one hour: anti-mouse (926–32212) (IRDye 800CW Donkey Anti-Mouse IgG (H + L)) or anti-rabbit (926–68073) (IRDye 680RD Donkey (polyclonal) Anti-Rabbit IgG (H + L), LI-COR Biosciences Inc., Lincoln, NE, USA) secondary antibodies. Signal was revealed with a LI-COR Odyssey Infrared Imaging System (LI-COR Biosciences Inc., Lincoln, NE, USA), analyzed and quantified using the free ImageJ software.

### Flow cytometry

Lungs were chopped to tiny pieces with scalpels and scissors. They were then treated with 10 mL of 0.1% Collagenase I and 0.25% Collagenase II (Worthington Biochemical Corporation, Lakewood, NJ, USA) in 4% FBS 1X PBS for 2 h at 37 °C / 5%CO_2_. Fully supplemented RPMI-1640 was added to deactivate the collagenase solution and cell suspensions were passed through cell strainers of 100 and then 40μm (SPL Life Sciences, Cat. No.: 93100 and 93040) in order to obtain single cell suspensions. Cells were also treated with red blood lysis buffer for 10 min at 4 °C to remove red blood cells. This method is described in^56^. Cells were then washed with PBS, and fixed and permeabilized with the BD Cytofix/Cytoperm^TM^ kit (Cat. No.: 554714 BD Biosciences). Ki67 marker was used to measure proliferation (#ab15580) (Abcam, Cambridge, UK). Cells were stained for α-SMA (#NBP2-34760PE) (1:200) (Novus Biologicals LLC, Centennial, CO, USA), desmin (#ab32362) (1:70) (Abcam, Cambridge, UK) and vimentin (#ab194719) (1:5’000) (Abcam, Cambridge, UK). Secondary antibodies were Goat anti-Rabbit (#P-10994) (1:100) (Thermo Fischer Scientific Inc., Reinach, Switzerland) (secondary antibody to desmin) and Goat anti-Rabbit (#ab150077) (1:2’000) (Abcam, Cambridge, UK) (secondary antibody to Ki67). Since both secondary antibodies were Goat anti-Rabbit, first cells were stained with Ki67 and Goat anti-Rabbit-Alexa 488. Then cells were washed and staining with α-SMA, vimentin and desmin followed. Finally, cells were washed and stained for goat anti-Rabbit-Pacific Blue. Cells were washed and flow cytometry analysis followed. Myofibroblasts were characterized as α-SMA^+^/vimentin^+^/desmin^−^. Cells that were α-SMA^+^/vimentin^−^/desmin^+^ were characterized as α-SMA cells. The entire staining procedure was done on ice. Single cell gate was set as the stopping gate and at least 30,000 events were acquired per sample. Data was analyzed on FlowJo software (Tri-Star). A representative gating strategy is shown on Supplementary Figure S1.

### Statistical analyses

Statistical analyses were done in GraphPad Prism software (GraphPad software Inc., La Jolla, CA, USA). Either two-way ANOVA (for respiratory function parameters and flow cytometry experiments) or Student’s t test for two by two comparisons (for Western blots and RT-qPCR experiments) were used.

## Supplementary information

Supplementary information

## Data Availability

All data generated or analyzed during this study are included in this published article.

## References

[1] Chiquet-Ehrismann R, Chiquet M (2003). Tenascins: regulation and putative functions during pathological stress. J. Pathol..

[2] Chiquet-Ehrismann R (2004). Tenascins. Int. J. Biochem. Cell Biol..

[3] Hsia HC, Schwarzbauer JE (2005). Meet the tenascins: multifunctional and mysterious. J. Biol. Chem..

[4] Midwood KS, Hussenet T, Langlois B, Orend G (2011). Advances in tenascin-C biology. Cell. Mol. life sciences: CMLS.

[5] Midwood KS, Chiquet M, Tucker RP, Orend G (2016). Tenascin-C at a glance. J. Cell Sci..

[6] Giblin SP, Midwood KS (2015). Tenascin-C: Form versus function. Cell Adh Migr..

[7] Chiquet-Ehrismann R, Tucker RP (2004). Connective tissues: signalling by tenascins. Int. J. Biochem. Cell Biol..

[8] Chiquet M (1992). Tenascin: an extracellular matrix protein involved in morphogenesis of epithelial organs. Kidney Int..

[9] Young SL, Chang LY, Erickson HP (1994). Tenascin-C in rat lung: distribution, ontogeny and role in branching morphogenesis. Dev. Biol..

[10] Midwood KS, Orend G (2009). The role of tenascin-C in tissue injury and tumorigenesis. J. Cell Commun. Signal..

[11] Chiquet-Ehrismann R, Orend G, Chiquet M, Tucker RP, Midwood KS (2014). Tenascins in stem cell niches. Matrix Biol..

[12] Laitinen A (1997). Tenascin is increased in airway basement membrane of asthmatics and decreased by an inhaled steroid. Am. J. Respir. Crit. Care Med..

[13] Estany S (2014). Lung fibrotic tenascin-C upregulation is associated with other extracellular matrix proteins and induced by TGFbeta1. BMC Pulm. Med..

[14] Kaarteenaho-Wiik R (2002). Tenascin-C is highly expressed in respiratory distress syndrome and bronchopulmonary dysplasia. J. Histochem. Cytochem..

[15] Lofdahl M, Kaarteenaho R, Lappi-Blanco E, Tornling G, Skold MC (2011). Tenascin-C and alpha-smooth muscle actin positive cells are increased in the large airways in patients with COPD. Respir. Res..

[16] Yasuda M (2018). Characterization of tenascin-C as a novel biomarker for asthma: utility of tenascin-C in combination with periostin or immunoglobulin E. Allergy Asthma Clin. Immunol..

[17] Saga Y, Yagi T, Ikawa Y, Sakakura T, Aizawa S (1992). Mice develop normally without tenascin. Genes. Dev..

[18] Forsberg E (1996). Skin wounds and severed nerves heal normally in mice lacking tenascin-C. Proc. Natl Acad. Sci. U S Am..

[19] Carey WA, Taylor GD, Dean WB, Bristow JD (2010). Tenascin-C deficiency attenuates TGF-ss-mediated fibrosis following murine lung injury. Am. J. Physiol. Lung Cell Mol. Physiol.

[20] Snyder JC, Zemke AC, Stripp BR (2009). Reparative capacity of airway epithelium impacts deposition and remodeling of extracellular matrix. Am. J. Respir. Cell Mol. Biol..

[21] Nakahara H (2006). Deficiency of tenascin C attenuates allergen-induced bronchial asthma in the mouse. Eur. J. immunology.

[22] Roth-Kleiner M, Hirsch E, Schittny JC (2004). Fetal lungs of tenascin-C-deficient mice grow well, but branch poorly in organ culture. Am. J. Respir. Cell Mol. Biol..

[23] Mund, S. I. & Schittny, J. C. Tenascin-C deficiency impairs alveolarization and microvascular maturation during postnatal lung development. *J Appl Physiol*, in press (2020). 10.1152/japplphysiol.00258.2019 PMC7272747 32078464

[24] Midwood K (2009). Tenascin-C is an endogenous activator of Toll-like receptor 4 that is essential for maintaining inflammation in arthritic joint disease. Nat. Med..

[25] Piccinini AM, Zuliani-Alvarez L, Lim JM, Midwood KS (2016). Distinct microenvironmental cues stimulate divergent TLR4-mediated signaling pathways in macrophages. Sci. Signal..

[26] Trebaul A, Chan EK, Midwood KS (2007). Regulation of fibroblast migration by tenascin-C. Biochem. Soc. Trans..

[27] Chiovaro F, Chiquet-Ehrismann R, Chiquet M (2015). Transcriptional regulation of tenascin genes. Cell Adh Migr..

[28] Mizikova I, Morty RE (2015). The Extracellular Matrix in Bronchopulmonary Dysplasia: Target and Source. Front. Med..

[29] Prakash YS (2013). Airway smooth muscle in airway reactivity and remodeling: what have we learned?. Am. J. Physiol. Lung Cell Mol. Physiol.

[30] Gosens R, Grainge C (2015). Bronchoconstriction and airway biology: potential impact and therapeutic opportunities. Chest.

[31] Prakash YS (2016). Emerging concepts in smooth muscle contributions to airway structure and function: implications for health and disease. Am. J. Physiol. Lung Cell Mol. Physiol.

[32] Copland IB (2004). High tidal volume ventilation causes different inflammatory responses in newborn versus adult lung. Am. J. Respir. Crit. Care Med..

[33] Morrisey EE, Hogan BL (2010). Preparing for the first breath: genetic and cellular mechanisms in lung development. Dev. Cell.

[34] El Agha E, Bellusci S (2014). Walking along the Fibroblast Growth Factor 10 Route: A Key Pathway to Understand the Control and Regulation of Epithelial and Mesenchymal Cell-Lineage Formation during Lung Development and Repair after Injury. Scientifica.

[35] Cohen ED (2009). Wnt signaling regulates smooth muscle precursor development in the mouse lung via a tenascin C/PDGFR pathway. J. Clin. Invest..

[36] Gu L (2007). Effect of TGF-beta/Smad signaling pathway on lung myofibroblast differentiation. Acta Pharmacol. Sin..

[37] Fitch PM, Howie SE, Wallace WA (2011). Oxidative damage and TGF-beta differentially induce lung epithelial cell sonic hedgehog and tenascin-C expression: implications for the regulation of lung remodelling in idiopathic interstitial lung disease. Int. J. Exp. Pathol..

[38] Morty RE, Konigshoff M, Eickelberg O (2009). Transforming growth factor-beta signaling across ages: from distorted lung development to chronic obstructive pulmonary disease. Proc. Am. Thorac. Soc..

[39] Verrecchia F, Chu ML, Mauviel A (2001). Identification of novel TGF-beta /Smad gene targets in dermal fibroblasts using a combined cDNA microarray/promoter transactivation approach. J. Biol. Chem..

[40] Jinnin M (2004). Tenascin-C upregulation by transforming growth factor-beta in human dermal fibroblasts involves Smad3, Sp1, and Ets1. Oncogene.

[41] Doerner AM, Zuraw BL (2009). TGF-beta1 induced epithelial to mesenchymal transition (EMT) in human bronchial epithelial cells is enhanced by IL-1beta but not abrogated by corticosteroids. Respir. Res..

[42] Desai VD, Hsia HC, Schwarzbauer JE (2014). Reversible modulation of myofibroblast differentiation in adipose-derived mesenchymal stem cells. PLoS one.

[43] Bhattacharyya S (2016). Tenascin-C drives persistence of organ fibrosis. Nat. Commun..

[44] Tamaoki M (2005). Tenascin-C regulates recruitment of myofibroblasts during tissue repair after myocardial injury. Am. J. Pathol..

[45] Huang T, Schor SL, Hinck AP (2014). Biological activity differences between TGF-beta1 and TGF-beta3 correlate with differences in the rigidity and arrangement of their component monomers. Biochem..

[46] Zuliani-Alvarez L (2017). Mapping tenascin-C interaction with toll-like receptor 4 reveals a new subset of endogenous inflammatory triggers. Nat. Commun..

[47] Amin K (2000). Inflammation and structural changes in the airways of patients with atopic and nonatopic asthma. BHR Group. Am. J. Respir. Crit. Care Med..

[48] Karjalainen EM (2000). Evidence of airway inflammation and remodeling in ski athletes with and without bronchial hyperresponsiveness to methacholine. Am. J. Respir. Crit. Care Med..

[49] Doan TC (2018). Matrix protein tenascin-C expands and reversibly blocks maturation of murine eosinophil progenitors. J. Allergy Clin. Immunol..

[50] Jeffery PK (2001). Remodeling in asthma and chronic obstructive lung disease. Am. J. Respir. Crit. Care Med..

[51] Wilhelm CP, Chipps BE (2016). Bronchial thermoplasty: a review of the evidence. Ann. Allergy Asthma Immunol..

[52] Kwak HJ (2015). The Wnt/beta-catenin signaling pathway regulates the development of airway remodeling in patients with asthma. Exp. Mol. Med..

[53] Hussain M (2017). Wnt/beta-catenin signaling links embryonic lung development and asthmatic airway remodeling. Biochim. Biophys. Acta Mol. Basis Dis..

[54] Salazar E, Knowles JH (1964). An Analysis of Pressure-Volume Characteristics of the Lungs. J. Appl. Physiol..

[55] Joester A, Faissner A (1999). Evidence for combinatorial variability of tenascin-C isoforms and developmental regulation in the mouse central nervous system. J. Biol. Chem..

[56] Tamo L (2018). Gene Network Analysis of Interstitial Macrophages After Treatment with Induced Pluripotent Stem Cells Secretome (iPSC-cm) in the Bleomycin Injured Rat Lung. Stem Cell Rev..

