# Supplementary material for: Tenascin-C inactivation impacts lung structure and function beyond lung development
Source: Sci Rep. 2020 Mar 20;10:5118. doi: 10.1038/s41598-020-61919-x (PMC7083919; doi:10.1038/s41598-020-61919-x)
Supplement: Supplementary file 1 — Supplementary information [file 41598_2020_61919_MOESM1_ESM.pdf]

Tenascin-C inactivation impacts lung structure and function beyond lung development

Sandrine Gremlich[1*], Matthias Roth-Kleiner[1], Lucile Equey[1], Kleanthis Fytianos[2,3], Johannes C. Schittny[4], Tiziana P. Cremona[4]

[1]Clinic of Neonatology, Department woman-mother-child, University Hospital and University of Lausanne, Lausanne, Switzerland

[2]Department of Bio-medical Research, University of Bern, Bern, Switzerland

[3]Division of Pulmonary Medicine, University of Bern, Bern, Switzerland

[4]Institute of Anatomy, University of Bern, Bern, Switzerland

	forward	reverse
**TGFβ1**	5’-CTG CTG ACC CCC ACT GAT AC-3’	5’-AGC CCT GTA TTC CGT CTC CT-3’
**TGFβ2**	5’-AGA GCT CGA GGC GAG ATT TG-3’	5’-GAT GTG GGG TCT TCC CAC TG-3’
**TGFβ3**	5’-GGA CTT CGG CCA CAT CAA GA-3’	5’-ATA GGG GAC GTG GGT CAT CA-3’
**TGFβR1**	5’-GCA TTG GCA AAG GTC GGT TT-3’	5’-TGC CTC TCG GAA CCA TGA AC-3’
**TGFβR2**	5’-GTG AGA CTG TCC ACT TGC GA-3’	5’-TGT CGT TCT TCC TCC ACA CG-3’
**TGFβR3**	5’-CTG CCA AGG GAG GTT CAC AT-3’	5’-AGC AGG AAC ACA ACA GGC TT-3’
**Smad2**	5’-GTA TGG ACA CAG GCT CTC CG-3’	5’-ACC AGA ATG CAG GTT CCG AG-3’
**Smad3**	5’-CTC AAG AAG ACG GGG CAG TT-3’	5’-AGG CGG CAG TAG ATA ACG TG-3’
**TLR4**	5’-AGC CGG AAG GTT ATT GTG GT-3’	5’-CAG CAG GGA CTT CTC AAC CT-3’
**MyD88**	5’-CCG CCT ATC GCT GTT CTT GA-3’	5’-CCA GGC ATC CAA CAA ACT GC-3’

Supplementary Table SI: Primers panel for RT-qPCR.

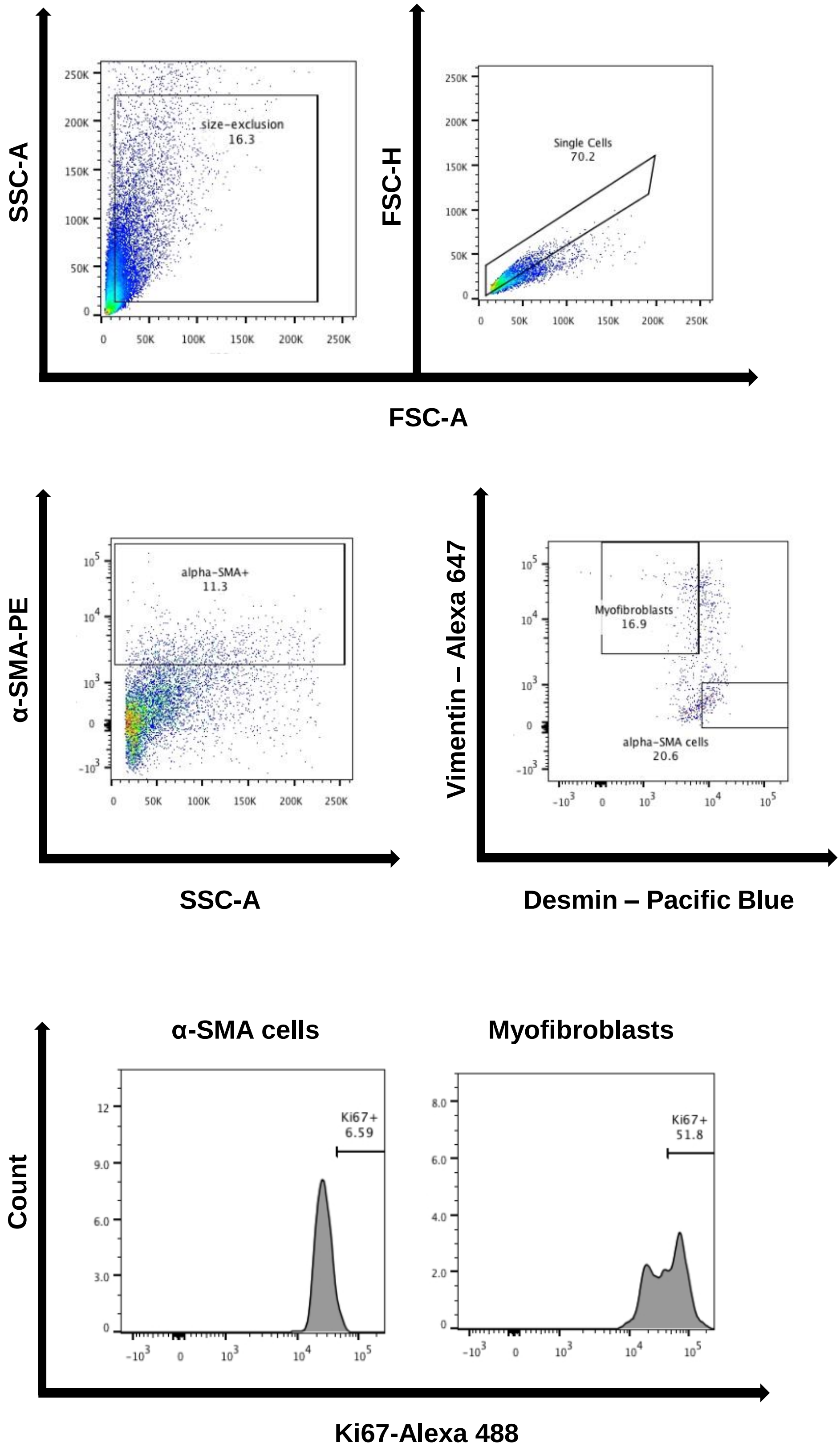

Supplementary Figure S1: Flow cytometry gating strategy. Representative dot plots are shown. The same gating was applied for all samples. SSC: side scatter. FSC: forward scatter. α-SMA-PE: alpha-smooth muscle actin-phycoerythrin.

Supplementary Figure S2:

a

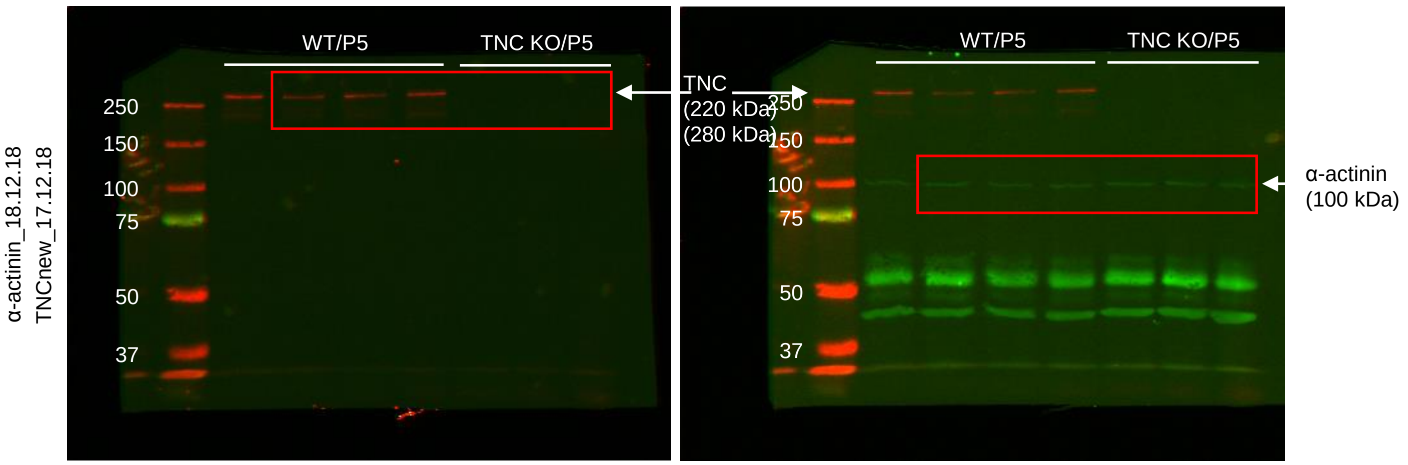

b

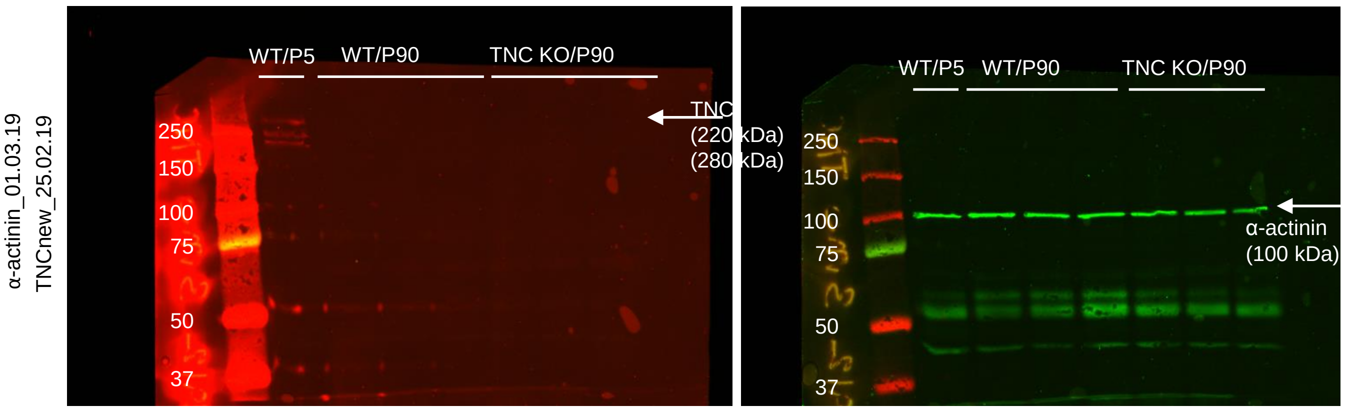

c

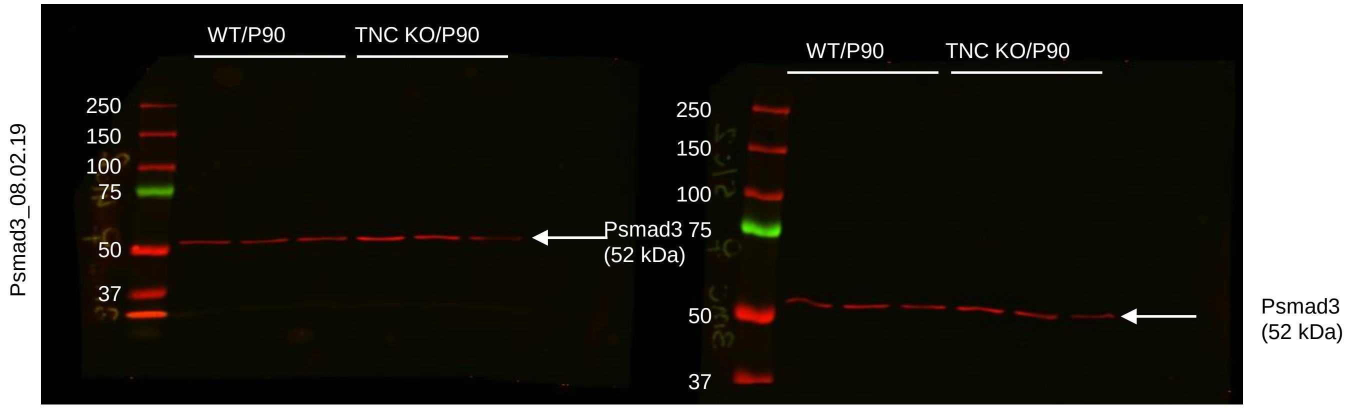

d

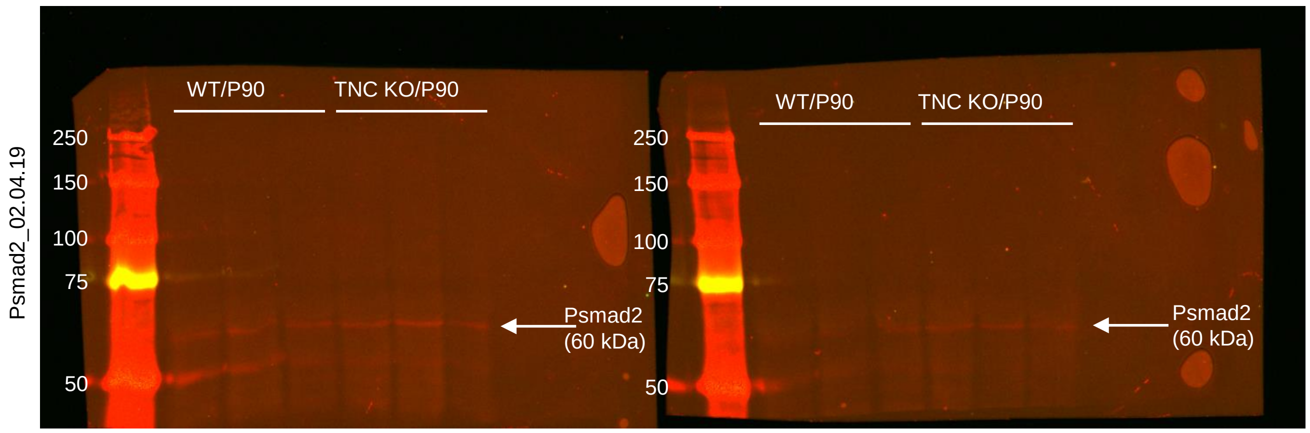

e

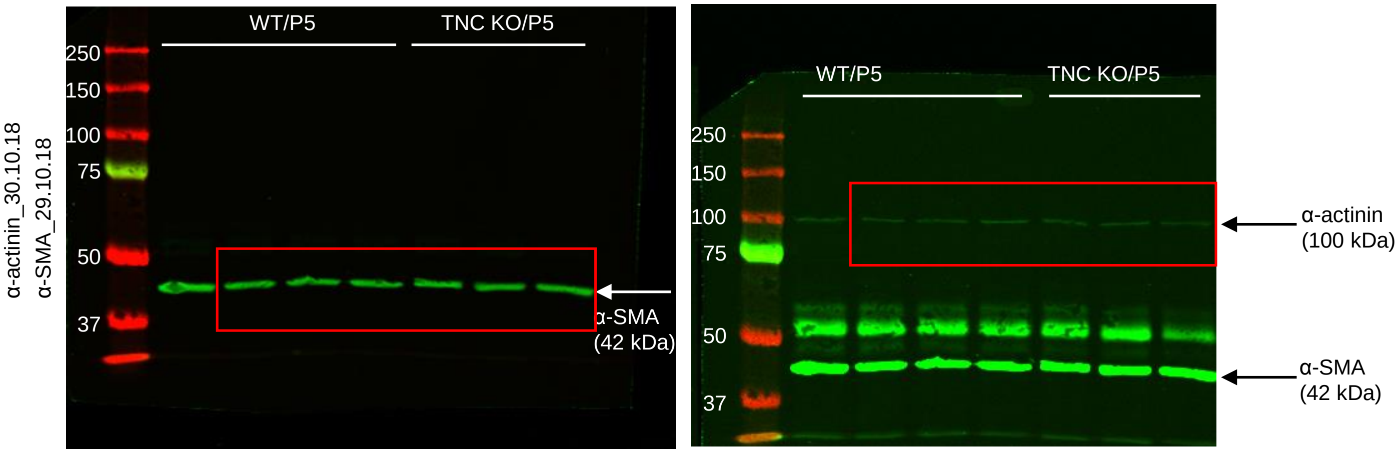

α-actinin_30.10.18
α-SMA_29.10.18
WT/P5
TNC KO/P5
250
150
100
75
50
37
α-SMA
(42 kDa)
WT/P5
TNC KO/P5
250
150
100
75
50
37
α-actinin
(100 kDa)
α-SMA
(42 kDa)

f

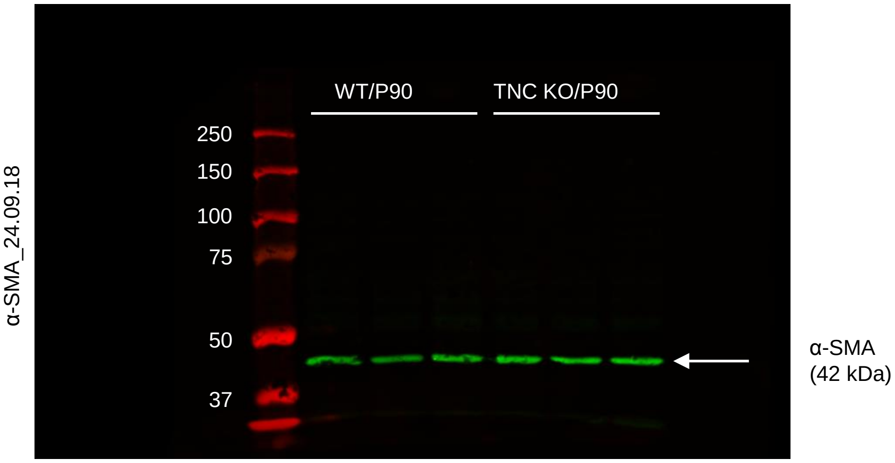

α-SMA_24.09.18
WT/P90
TNC KO/P90
250
150
100
75
50
37
α-SMA
(42 kDa)

g

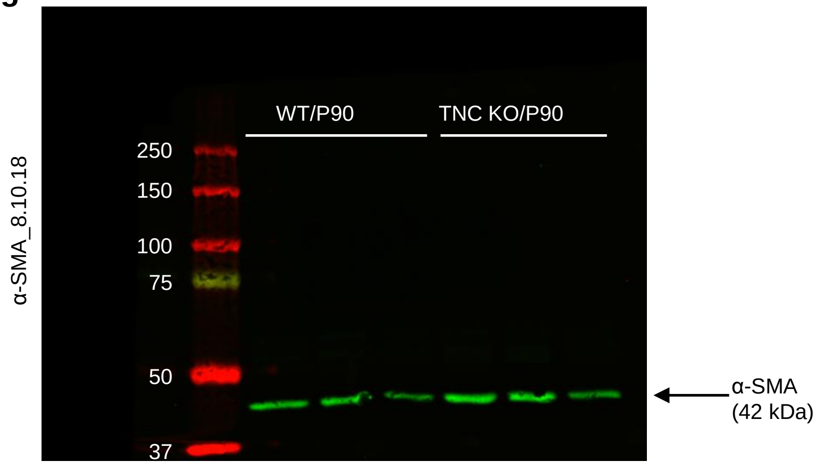

α-SMA_8.10.18
WT/P90
TNC KO/P90
250
150
100
75
50
37
α-SMA
(42 kDa)

h

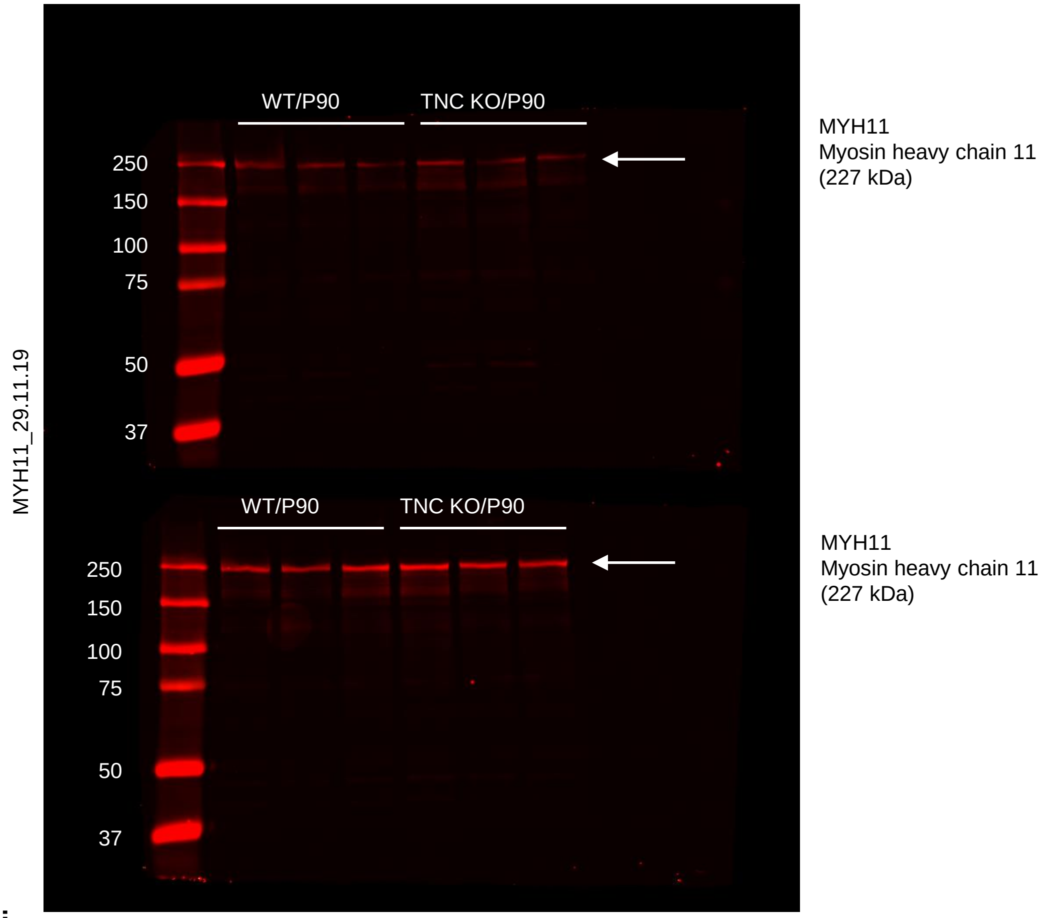
MYH11_29.11.19
WT/P90
TNC KO/P90
250
150
100
75
50
37
MYH11
Myosin heavy chain 11
(227 kDa)
WT/P90
TNC KO/P90
250
150
100
75
50
37
MYH11
Myosin heavy chain 11
(227 kDa)

i

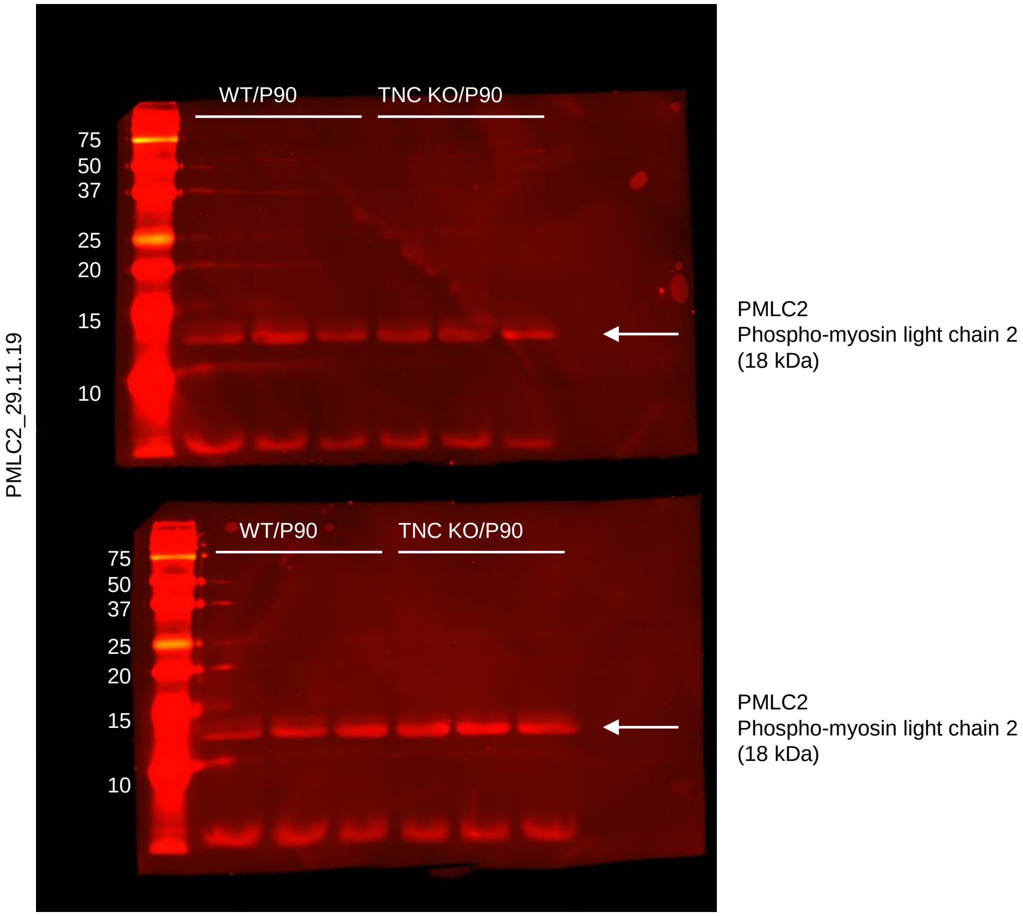
PMLC2_29.11.19
WT/P90
TNC KO/P90
75
50
37
25
20
15
10
PMLC2
Phospho-myosin light chain 2
(18 kDa)
WT/P90
TNC KO/P90
75
50
37
25
20
15
10
PMLC2
Phospho-myosin light chain 2
(18 kDa)

j

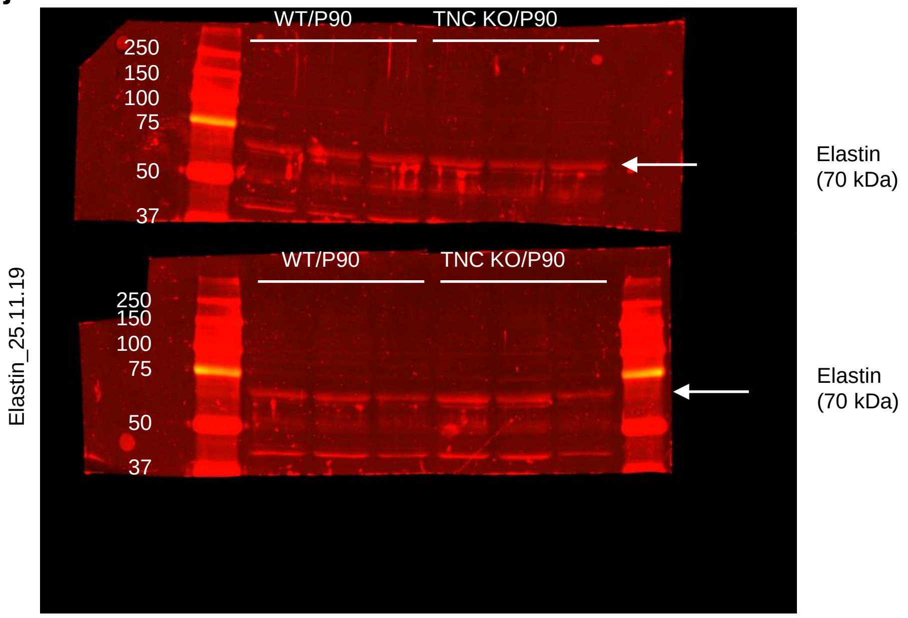

Elastin_25.11.19
WT/P90
TNC KO/P90
250
150
100
75
50
37
Elastin
(70 kDa)
WT/P90
TNC KO/P90
250
150
100
75
50
37
Elastin
(70 kDa)

k

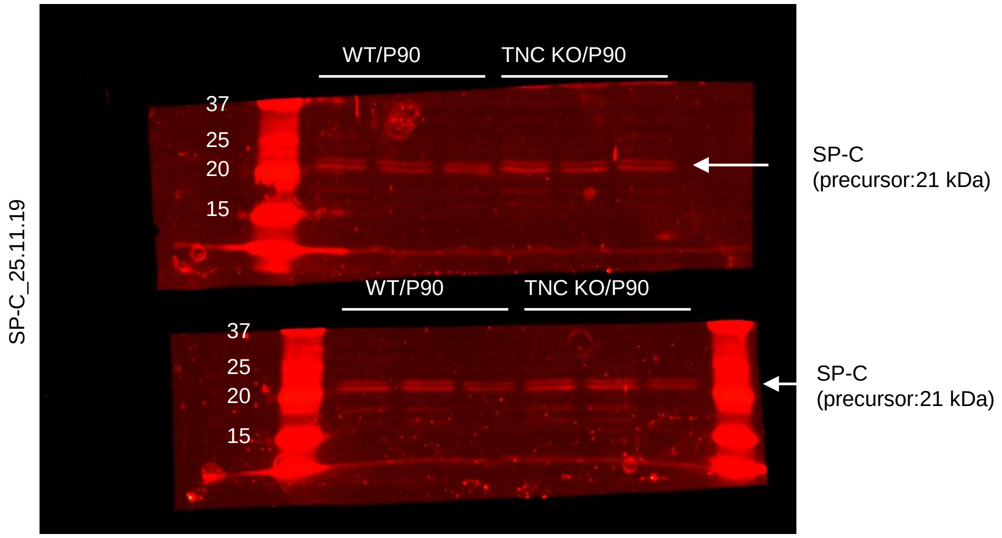

SP-C_25.11.19
WT/P90
TNC KO/P90
37
25
20
15
SP-C
(precursor:21 kDa)
WT/P90
TNC KO/P90
37
25
20
15
SP-C
(precursor:21 kDa)

l

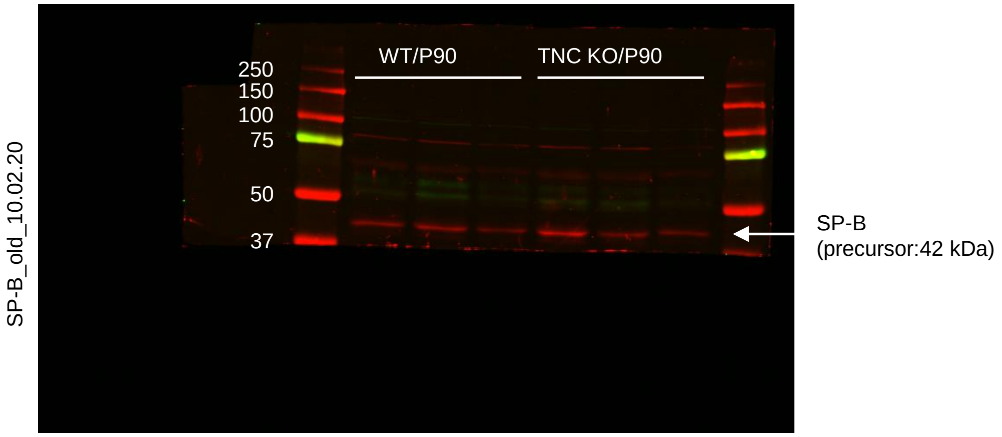

SP-B_old_10.02.20
WT/P90
TNC KO/P90
250
150
100
75
50
37
SP-B
(precursor:42 kDa)

m

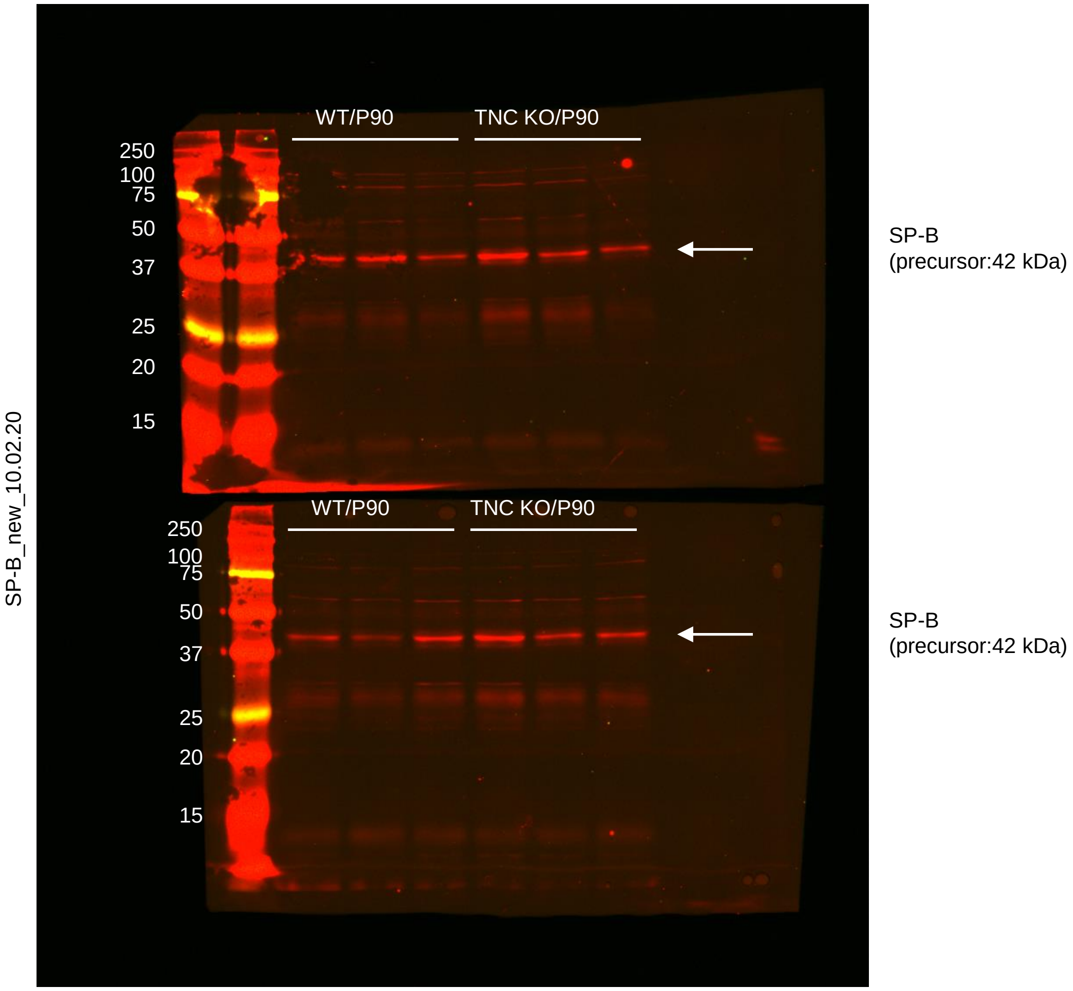

Supplementary Figure S2: Full length original Western blots. Panel a: full length blots for Figure 2e. Panel b: full length blots for Figure 2f. Panel c: full length blots for Figure 8d. Panel d: full length blots for Figure 8b. Panel e: full length blots for Figure 7g. Panel f: full length blots for Figure 7h. Panel g: other full length blots for Figure 7h. Panel h: full length blots for Figure 7l. Panel i: full length blots for Figure 7k. Panel j: full length blots for Figure 6j. Panel k: full length blots for Figure 4k. Panel l: full length blots for Figure 4k. Panel m: other full length blots for Figure 4k. α-actinin was used for normalization.
